# Supplementary material for: Modeling glioblastoma heterogeneity as a dynamic network of cell states
Source: Mol Syst Biol. 2021 Sep 16;17(9):e10105. doi: 10.15252/msb.202010105 (PMC8444284; doi:10.15252/msb.202010105)
Supplement: Supplementary file 5 — Source Data for Figure 3 [file MSB-17-e10105-s001.zip › Figure3A_sourcedata/GSEA_3065/hallmarks_state5.GseaPreranked.1623416302988/HALLMARK_UV_RESPONSE_DN.html]

Details for gene set HALLMARK\_UV\_RESPONSE\_DN[GSEA]

|  || Dataset | state5 |
| Phenotype | NoPhenotypeAvailable |
| Upregulated in class | na\_pos |
| GeneSet | HALLMARK\_UV\_RESPONSE\_DN |
| Enrichment Score (ES) | 0.32676387 |
| Normalized Enrichment Score (NES) | 1.3173198 |
| Nominal p-value | 0.012295082 |
| FDR q-value | 0.17408454 |
| FWER p-Value | 0.479 |
Table: GSEA Results Summary

  

Fig 1: Enrichment plot: HALLMARK\_UV\_RESPONSE\_DN      
 Profile of the Running ES Score & Positions of GeneSet Members on the Rank Ordered List

  

| PROBE | GENE SYMBOL | GENE\_TITLE | RANK IN GENE LIST | RANK METRIC SCORE | RUNNING ES | CORE ENRICHMENT || 1 | MMP16 |  |  | 165 | 0.470 | 0.0147 | Yes |
| 2 | CACNA1A |  |  | 176 | 0.460 | 0.0462 | Yes |
| 3 | LAMC1 |  |  | 382 | 0.348 | 0.0478 | Yes |
| 4 | LDLR |  |  | 404 | 0.339 | 0.0694 | Yes |
| 5 | COL5A2 |  |  | 407 | 0.337 | 0.0931 | Yes |
| 6 | ATP2B4 |  |  | 445 | 0.326 | 0.1120 | Yes |
| 7 | HAS2 |  |  | 462 | 0.322 | 0.1330 | Yes |
| 8 | CDK13 |  |  | 541 | 0.300 | 0.1455 | Yes |
| 9 | IGF1R |  |  | 557 | 0.295 | 0.1647 | Yes |
| 10 | PRKCA |  |  | 652 | 0.275 | 0.1736 | Yes |
| 11 | PMP22 |  |  | 663 | 0.272 | 0.1917 | Yes |
| 12 | NOTCH2 |  |  | 756 | 0.249 | 0.1990 | Yes |
| 13 | ATRN |  |  | 800 | 0.241 | 0.2113 | Yes |
| 14 | DLG1 |  |  | 924 | 0.221 | 0.2131 | Yes |
| 15 | ATP2B1 |  |  | 953 | 0.218 | 0.2254 | Yes |
| 16 | ATP2C1 |  |  | 957 | 0.217 | 0.2404 | Yes |
| 17 | ATXN1 |  |  | 1062 | 0.203 | 0.2431 | Yes |
| 18 | SYNE1 |  |  | 1079 | 0.201 | 0.2555 | Yes |
| 19 | SERPINE1 |  |  | 1221 | 0.182 | 0.2525 | Yes |
| 20 | TFPI |  |  | 1278 | 0.175 | 0.2586 | Yes |
| 21 | ERBB2 |  |  | 1309 | 0.170 | 0.2673 | Yes |
| 22 | MAGI2 |  |  | 1383 | 0.163 | 0.2706 | Yes |
| 23 | GJA1 |  |  | 1399 | 0.162 | 0.2804 | Yes |
| 24 | PRDM2 |  |  | 1407 | 0.161 | 0.2909 | Yes |
| 25 | SYNJ2 |  |  | 1418 | 0.160 | 0.3012 | Yes |
| 26 | PLPP3 |  |  | 1423 | 0.160 | 0.3120 | Yes |
| 27 | ARHGEF9 |  |  | 1463 | 0.156 | 0.3187 | Yes |
| 28 | PIK3R3 |  |  | 1584 | 0.142 | 0.3153 | Yes |
| 29 | SLC22A18 |  |  | 1593 | 0.142 | 0.3244 | Yes |
| 30 | FZD2 |  |  | 1701 | 0.133 | 0.3218 | Yes |
| 31 | DYRK1A |  |  | 1812 | 0.126 | 0.3183 | Yes |
| 32 | CDC42BPA |  |  | 1817 | 0.125 | 0.3268 | Yes |
| 33 | MET |  |  | 1992 | 0.112 | 0.3151 | No |
| 34 | BMPR1A |  |  | 2003 | 0.111 | 0.3219 | No |
| 35 | SCAF8 |  |  | 2171 | 0.100 | 0.3101 | No |
| 36 | PTEN |  |  | 2191 | 0.099 | 0.3150 | No |
| 37 | DMAC2L |  |  | 2342 | 0.089 | 0.3044 | No |
| 38 | KALRN |  |  | 2434 | 0.083 | 0.3001 | No |
| 39 | SLC7A1 |  |  | 2527 | 0.077 | 0.2952 | No |
| 40 | ADORA2B |  |  | 2563 | 0.076 | 0.2966 | No |
| 41 | TENT4A |  |  | 2603 | 0.073 | 0.2974 | No |
| 42 | ACVR2A |  |  | 2674 | 0.070 | 0.2945 | No |
| 43 | RASA2 |  |  | 2689 | 0.069 | 0.2978 | No |
| 44 | NIPBL |  |  | 2726 | 0.067 | 0.2985 | No |
| 45 | SCHIP1 |  |  | 2911 | 0.057 | 0.2818 | No |
| 46 | COL11A1 |  |  | 2950 | 0.055 | 0.2814 | No |
| 47 | INSIG1 |  |  | 3055 | 0.049 | 0.2732 | No |
| 48 | EFEMP1 |  |  | 3073 | 0.048 | 0.2747 | No |
| 49 | VAV2 |  |  | 3172 | 0.043 | 0.2667 | No |
| 50 | IGFBP5 |  |  | 3230 | 0.040 | 0.2631 | No |
| 51 | PHF3 |  |  | 3435 | 0.030 | 0.2423 | No |
| 52 | MAPK14 |  |  | 3545 | 0.025 | 0.2318 | No |
| 53 | SIPA1L1 |  |  | 3562 | 0.025 | 0.2317 | No |
| 54 | NRP1 |  |  | 3735 | 0.019 | 0.2137 | No |
| 55 | VLDLR |  |  | 3854 | 0.014 | 0.2014 | No |
| 56 | SMAD3 |  |  | 3915 | 0.011 | 0.1954 | No |
| 57 | ATRX |  |  | 3933 | 0.010 | 0.1941 | No |
| 58 | KCNMA1 |  |  | 4020 | 0.006 | 0.1849 | No |
| 59 | MTA1 |  |  | 4076 | 0.004 | 0.1790 | No |
| 60 | ZMIZ1 |  |  | 4395 | -0.010 | 0.1439 | No |
| 61 | RXRA |  |  | 4441 | -0.012 | 0.1397 | No |
| 62 | SDC2 |  |  | 4596 | -0.018 | 0.1236 | No |
| 63 | MYC |  |  | 4656 | -0.020 | 0.1184 | No |
| 64 | BCKDHB |  |  | 4744 | -0.023 | 0.1102 | No |
| 65 | BDNF |  |  | 4833 | -0.027 | 0.1022 | No |
| 66 | MIOS |  |  | 4880 | -0.028 | 0.0991 | No |
| 67 | AMPH |  |  | 4940 | -0.031 | 0.0946 | No |
| 68 | RUNX1 |  |  | 4949 | -0.031 | 0.0959 | No |
| 69 | TGFBR2 |  |  | 5140 | -0.038 | 0.0772 | No |
| 70 | NFKB1 |  |  | 5183 | -0.039 | 0.0753 | No |
| 71 | APBB2 |  |  | 5184 | -0.039 | 0.0781 | No |
| 72 | YTHDC1 |  |  | 5276 | -0.043 | 0.0709 | No |
| 73 | SFMBT1 |  |  | 5346 | -0.047 | 0.0664 | No |
| 74 | IRS1 |  |  | 5518 | -0.054 | 0.0510 | No |
| 75 | RND3 |  |  | 5668 | -0.061 | 0.0385 | No |
| 76 | MRPS31 |  |  | 5701 | -0.062 | 0.0393 | No |
| 77 | ICA1 |  |  | 5718 | -0.063 | 0.0419 | No |
| 78 | MAP2K5 |  |  | 5719 | -0.063 | 0.0463 | No |
| 79 | BHLHE40 |  |  | 5950 | -0.072 | 0.0255 | No |
| 80 | ADD3 |  |  | 5984 | -0.073 | 0.0270 | No |
| 81 | CDKN1B |  |  | 5996 | -0.074 | 0.0310 | No |
| 82 | DUSP1 |  |  | 5997 | -0.074 | 0.0362 | No |
| 83 | CAP2 |  |  | 6013 | -0.075 | 0.0398 | No |
| 84 | NR3C1 |  |  | 6073 | -0.078 | 0.0387 | No |
| 85 | PDLIM5 |  |  | 6127 | -0.081 | 0.0385 | No |
| 86 | FHL2 |  |  | 6169 | -0.083 | 0.0398 | No |
| 87 | AKT3 |  |  | 6191 | -0.084 | 0.0434 | No |
| 88 | NR1D2 |  |  | 6487 | -0.101 | 0.0173 | No |
| 89 | RBPMS |  |  | 6497 | -0.101 | 0.0234 | No |
| 90 | TJP1 |  |  | 6500 | -0.101 | 0.0304 | No |
| 91 | AGGF1 |  |  | 6522 | -0.103 | 0.0353 | No |
| 92 | FYN |  |  | 6823 | -0.123 | 0.0102 | No |
| 93 | SPOP |  |  | 6824 | -0.123 | 0.0189 | No |
| 94 | MGLL |  |  | 7100 | -0.145 | -0.0018 | No |
| 95 | NFIB |  |  | 7111 | -0.146 | 0.0074 | No |
| 96 | CITED2 |  |  | 7120 | -0.147 | 0.0169 | No |
| 97 | DDAH1 |  |  | 7257 | -0.162 | 0.0131 | No |
| 98 | CELF2 |  |  | 7440 | -0.185 | 0.0057 | No |
| 99 | ANXA4 |  |  | 7565 | -0.199 | 0.0059 | No |
| 100 | CAV1 |  |  | 7575 | -0.200 | 0.0191 | No |
| 101 | PRKAR2B |  |  | 7651 | -0.211 | 0.0256 | No |
| 102 | MAP1B |  |  | 7666 | -0.213 | 0.0391 | No |
| 103 | ID1 |  |  | 7821 | -0.238 | 0.0386 | No |
| 104 | SRI |  |  | 8709 | -0.572 | -0.0208 | No |
| 105 | ANXA2 |  |  | 8868 | -0.729 | 0.0131 | No |
Table: GSEA details [plain text format]

  

Fig 2: HALLMARK\_UV\_RESPONSE\_DN: Random ES distribution      
 Gene set null distribution of ES for **HALLMARK\_UV\_RESPONSE\_DN**
